# Supplementary material for: Constructing sulfur and oxygen super-coordinated main-group electrocatalysts for selective and cumulative H2O2 production
Source: Nat Commun. 2024 Jan 2;15:193. doi: 10.1038/s41467-023-44585-1 (PMC10761824; doi:10.1038/s41467-023-44585-1)
Supplement: Supplementary file 1 — Supplementary Information [file 41467_2023_44585_MOESM1_ESM.pdf]

Supplementary Information

Constructing sulfur and oxygen super-coordinated main-group electrocatalysts for selective and cumulative H_2O_2 production

Xiao Zhou[1†], Yuan Min[1†], Changming Zhao[2†], Cai Chen[2], Ming-Kun Ke[1], Shi-Lin Xu[1], Jie-Jie Chen[1], Yuen Wu[2*] and Han-Qing Yu[1*]

[1]CAS Key Laboratory of Urban Pollutant Conversion, Department of Environmental Science and Engineering, University of Science and Technology of China, Hefei 230026, China

[2]School of Chemistry and Materials Science, University of Science and Technology of China, Hefei 230026, China

Supporting Figures and Tables

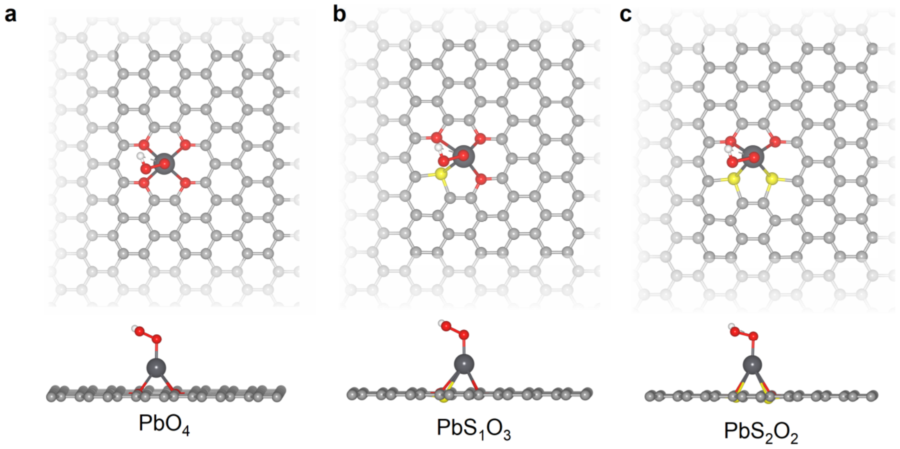

Supplementary Fig. 1 | Top and side views of *OOH species adsorbed on (a) PbO_4, (b) PbS_1O_3 and (c) PbS_2O_2. Gray, yellow, red, dark and white balls represent C, S, O, Pb and H atoms, respectively.

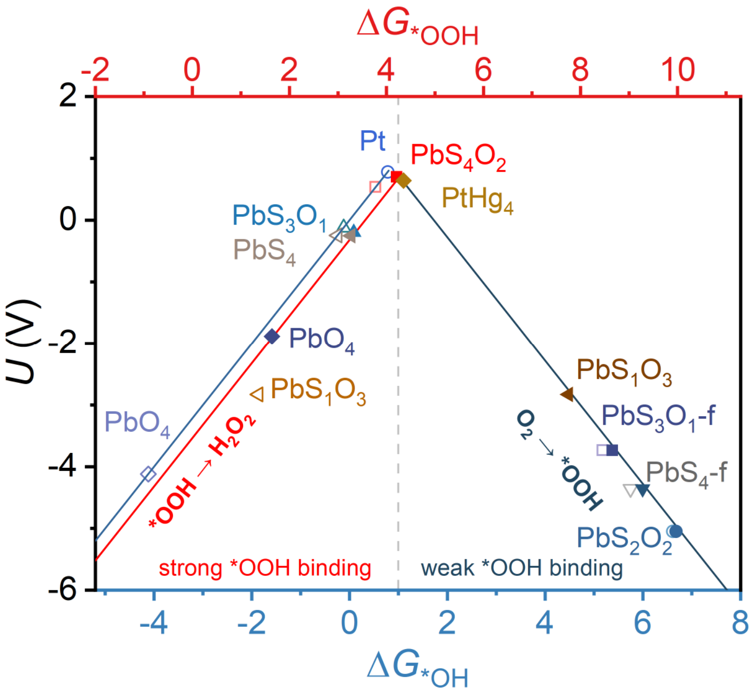

Supplementary Fig. 2 | Volcano plots for the 2e⁻ and 4e⁻ ORR on various Pb SA/OSC catalysts. The limiting potential was plotted as a function of ΔG_{*OH} (blue horizontal axis with open symbol) and ΔG_{*OOH} (red horizontal axis with solid symbol), showing the strongly and weakly bound *OOH regions for the 2e⁻ ORR process.

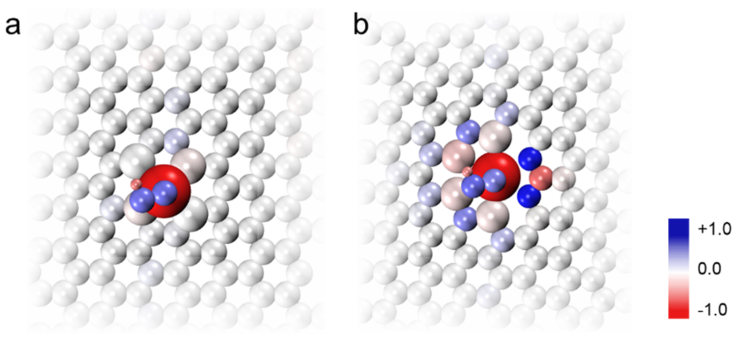

Supplementary Fig. 3 | Bader charge analysis of the adsorbed *OOH on the Pb site. (a) PbS_4 and (b) PbS_4O_2. The color of each atom indicates the Bader charge in units of elementary charge ($|e^-| = 1.6022 \times 10^{-19}$ C), with red and blue areas representing a loss and gain of electrons, respectively.

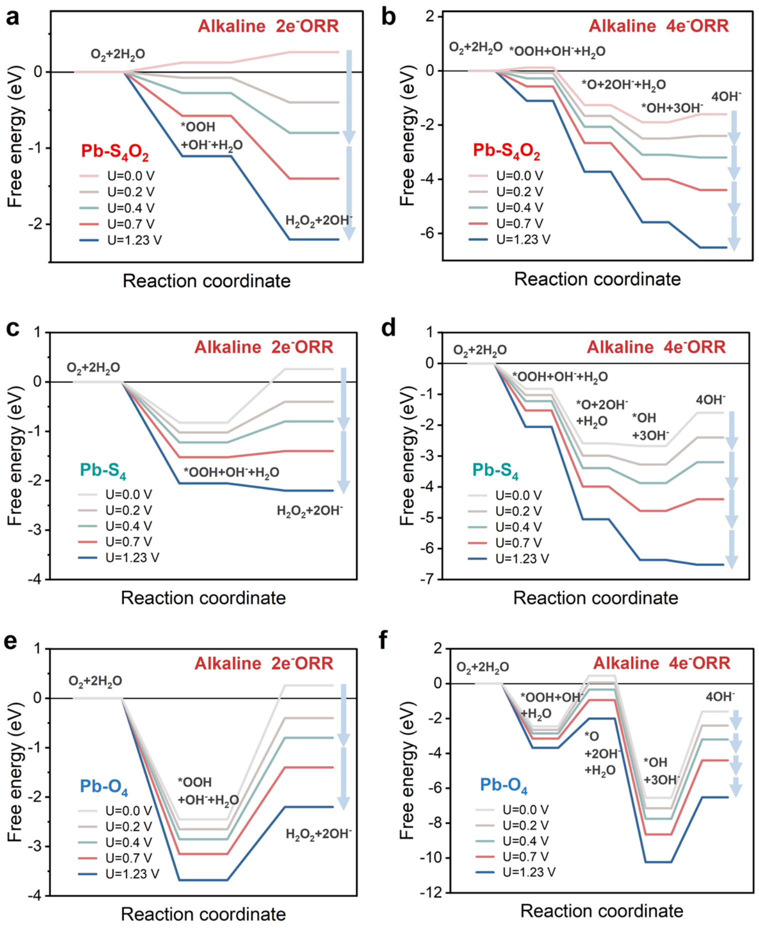

Supplementary Fig. 4 | Free-energy diagrams of 2e⁻ and 4e⁻ pathways for oxygen reduction. (a, b) PbS_4O_2, (c, d) PbS_4 and (e, f) PbO_4 catalysts in an alkaline medium at zero cell potential (U = 0) and the other potentials (U = 0.2, 0.4, 0.7 and 1.23 V).

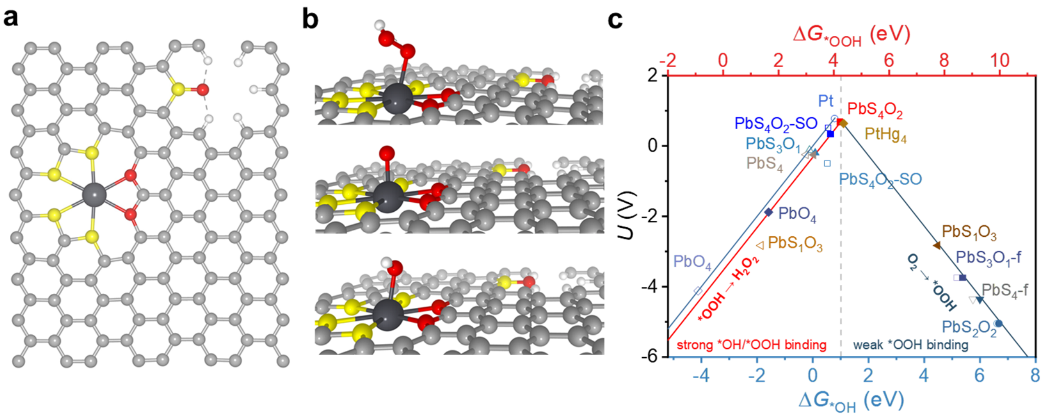

Supplementary Fig. 5 | Effect of the C–SO$_x$ group on the oxygen reduction reaction. (a) DFT-optimized geometry of the C–SO$_x$ group on the carbon support, denoted as PbS_4O_2-SO. (b) Intermediates geometries for the 2e$^-$ORR pathway of PbS_4O_2-SO. (c) Volcano plots for the 2e$^-$ and 4e$^-$ ORR on various Pb SA/OSC. The effect of oxidized sulfur (C–SO$_x$) groups on the PbS_4O_2 center was considered. A hexagonal ring structure of oxidized sulfur was selected as a model to conduct DFT calculations. Given the larger spatial distance between C–SO$_x$ group and Pb site, their interactions become weaker. To investigate the potential impact of C–SO$_x$ group on catalytic activity, only one benzene ring was employed to separate S and Pb. Based on a set of intermediates (*OOH, *O and *OH), the calculated overpotential for 2e$^-$ORR pathway (pH = 0, U = 0 V) was 0.35 V, suggesting the C–SO$_x$ groups are unfavorable for the formation of H_2O_2.

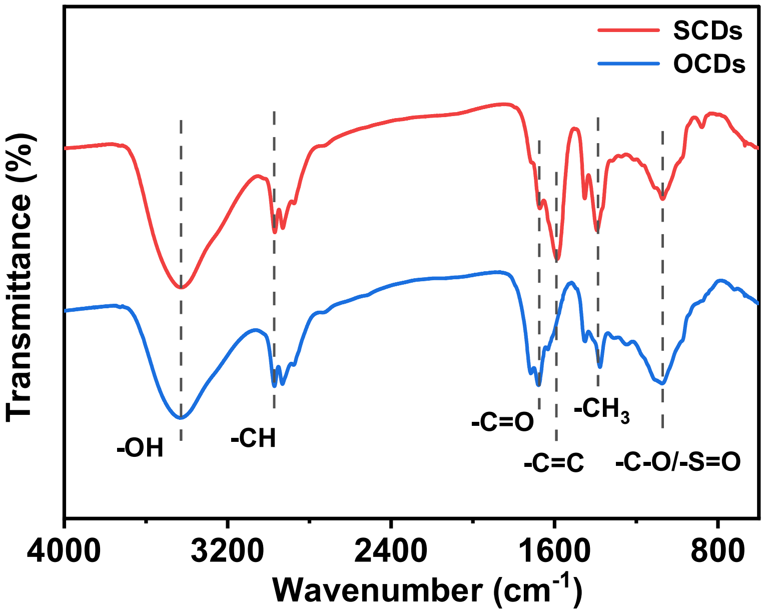

Supplementary Fig. 6 | Structural characterizations of carbon dots. FT-IR spectra of the oxygen-doped (OCDs) and sulfur-doped (SCDs) carbon dots.

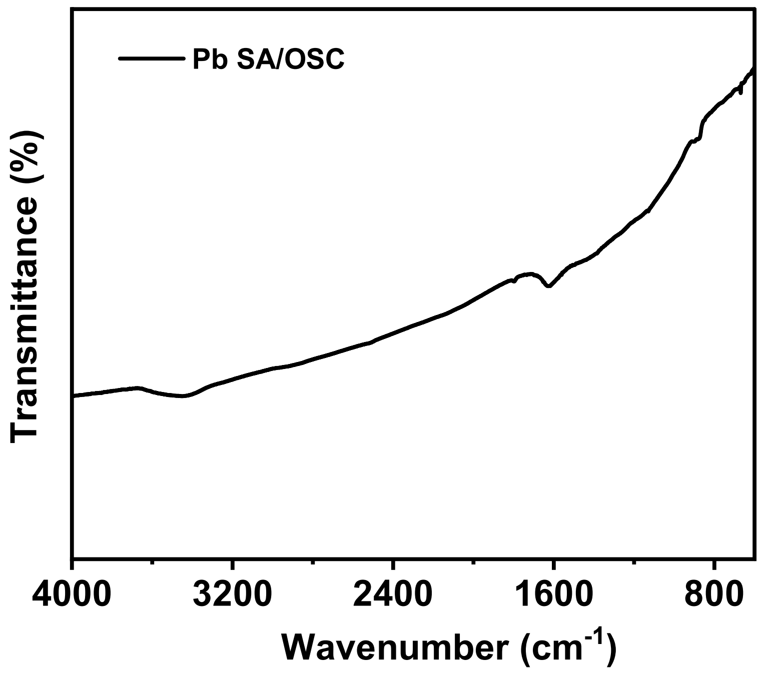

Supplementary Fig. 7 | Structural characterization of the prepared sample. FT-IR spectrum of Pb SA/OSC.

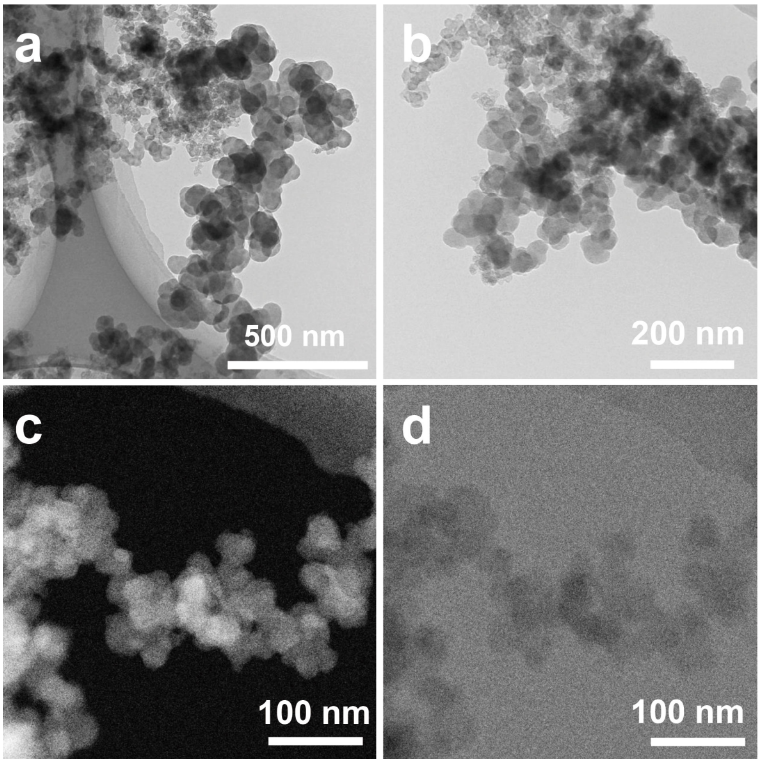

Supplementary Fig. 8 | Morphology characterizations of Pb SA/OSC. (a, b) TEM images of Pb SA/OSC at different magnifications. (c, d) HAADF-STEM images of Pb SA/OSC.

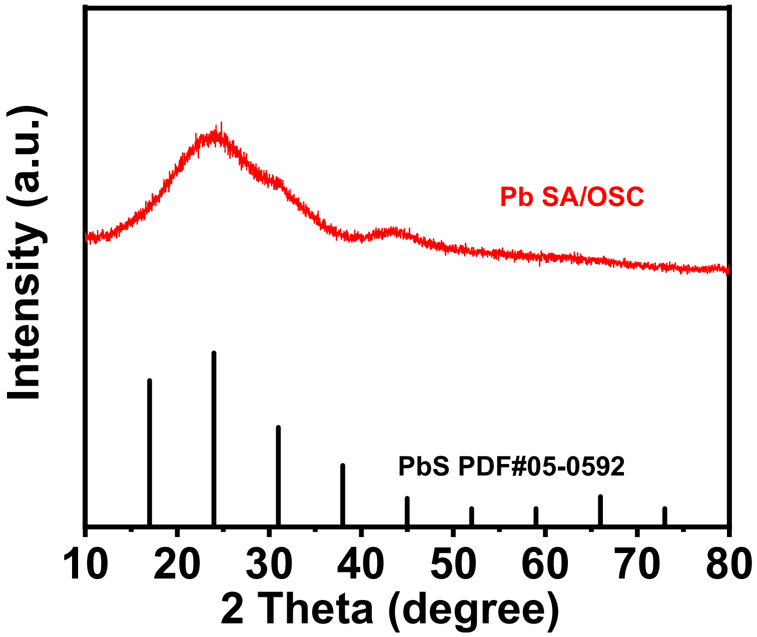

Supplementary Fig. 9 | XRD pattern of Pb SA/OSC sample. PDF#05-0592 is the JCPDS (Joint Committee on Powder Diffraction Standards) card number of PbS.

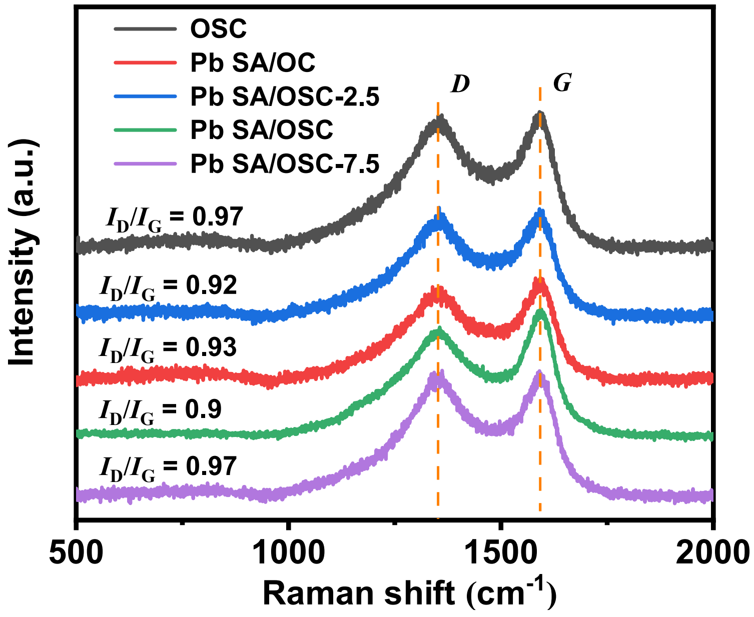

Supplementary Fig. 10 | Raman spectra of SCDs, Pb SA/OC, Pb SA/OSC, Pb SA/OSC-2.5 and Pb SA/OSC-7.5 samples. The intensity ratios (I_D/I_G) of the D-band (disordered/defective carbon) to G-band (graphitic carbon) were calculated.

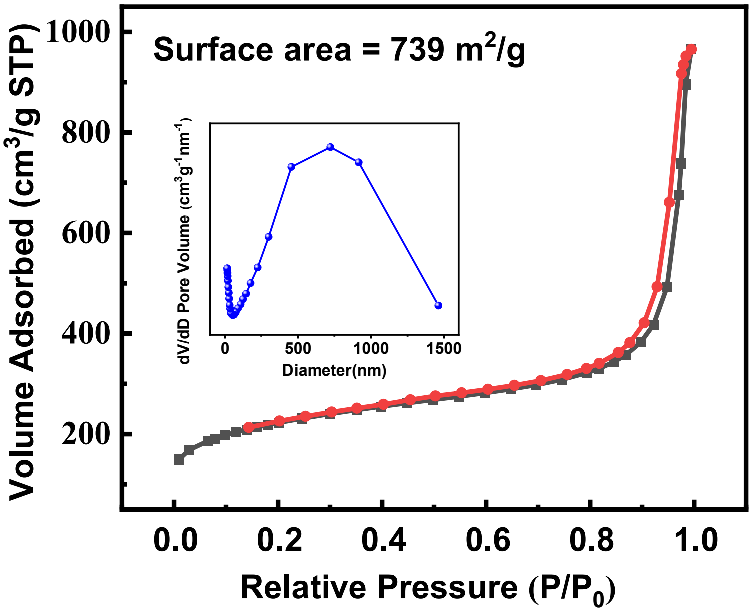

Supplementary Fig. 11 | BET surface area and pore size distribution. N_2 adsorption-desorption isotherms and pore-size distribution of Pb SA/OSC.

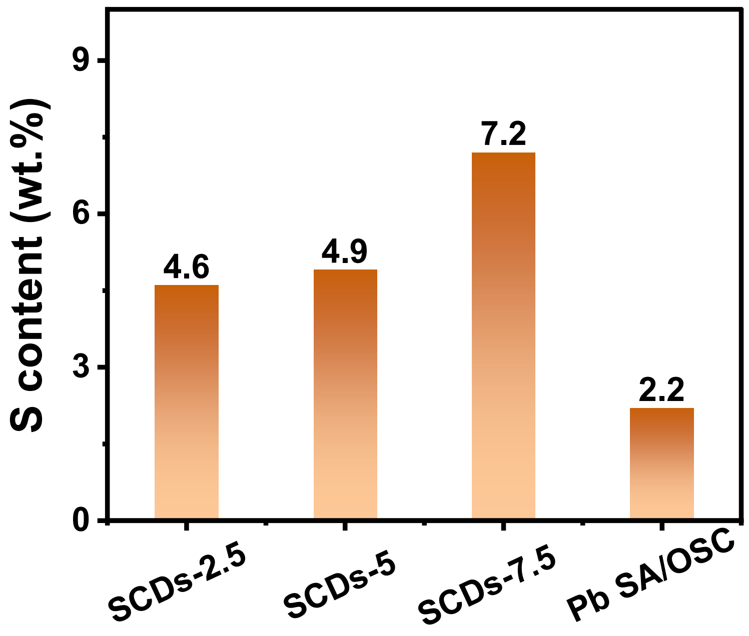

Supplementary Fig. 12 | Element analysis of sulfur in Pb SA/OSC and SCDs samples. The S contents of different SCDs samples increased with the rising amount of S-containing precursor.

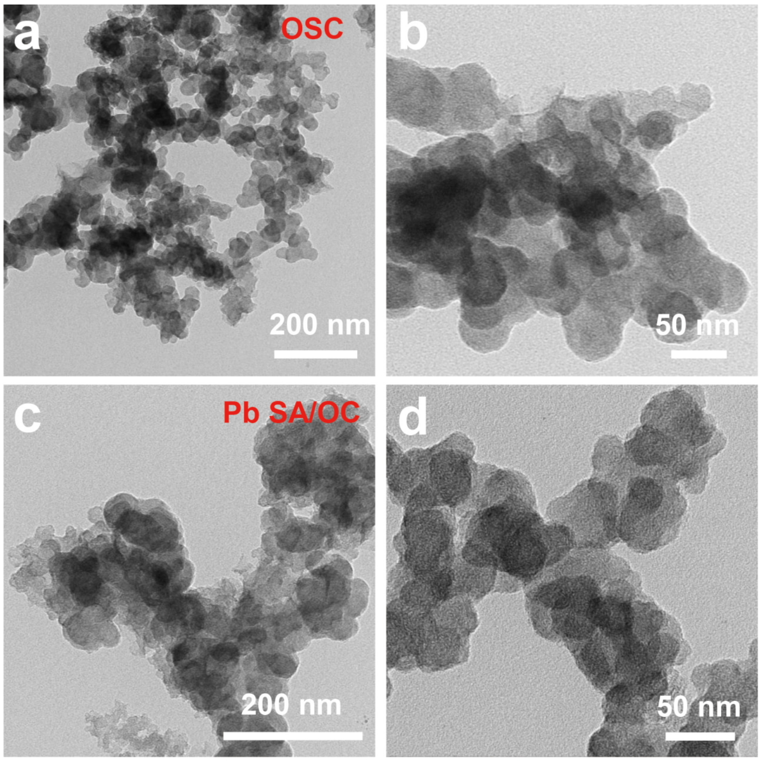

Supplementary Fig. 13 | Morphology characterizations of OSC and Pb SA/OC. (a, b) TEM images of OSC at different magnifications. (c, d) TEM images of Pb SA/OC at different magnifications.

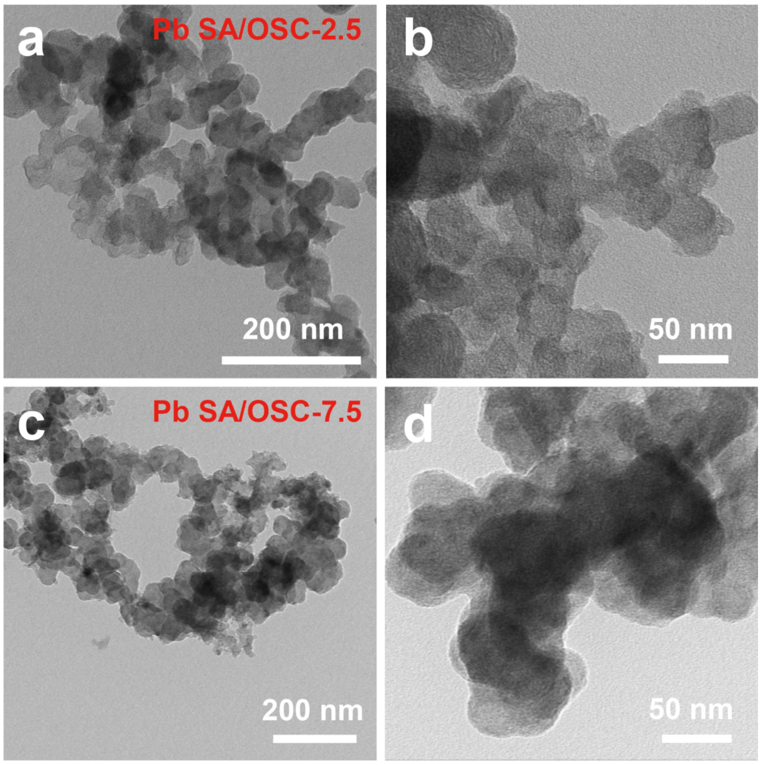

Supplementary Fig. 14 | Morphology characterizations of Pb SA/OSC-2.5 and Pb SA/OSC-7.5. (a, b) TEM images of Pb SA/OSC-2.5 at different magnifications. (c, d) TEM images of Pb SA/OSC-7.5 at different magnifications.

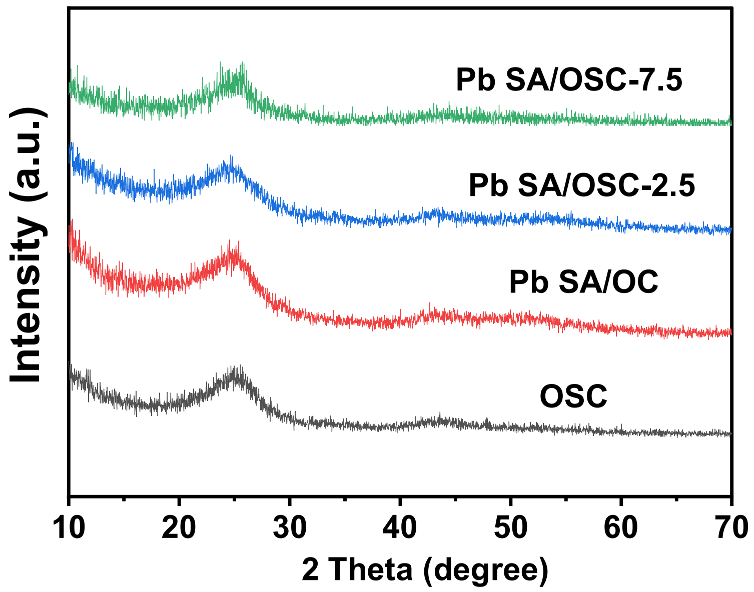

Supplementary Fig. 15 | Crystalline characterizations of the prepared samples. XRD patterns of OSC, Pb SA/OC, Pb SA/OSC-2.5 and Pb SA/OSC-7.5 samples.

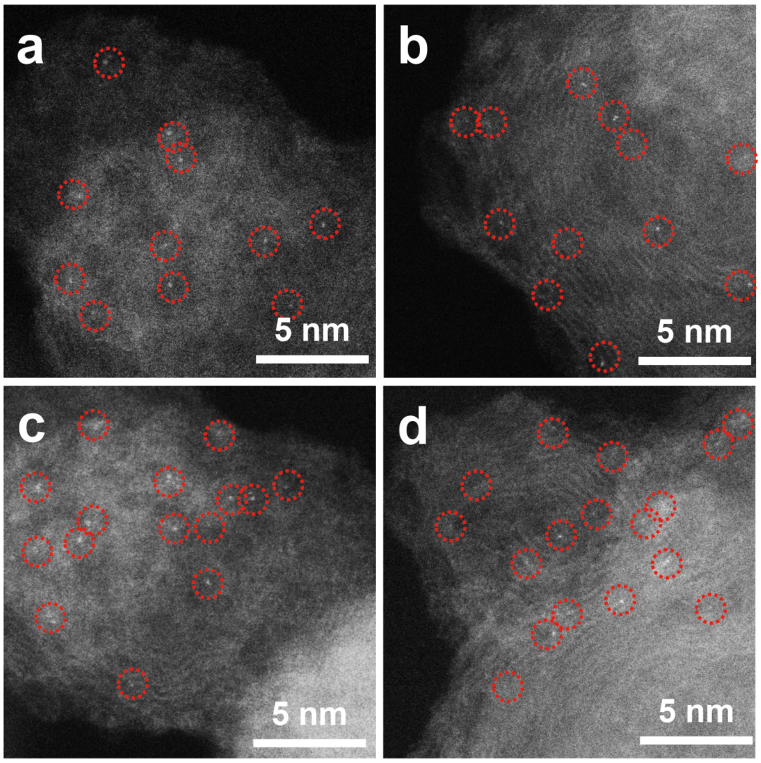

Supplementary Fig. 16 | Aberration-corrected HAADF-STEM characterization of Pb SA/OSC. Four representative HAADF-STEM images of Pb SA/OSC at different areas (a-d).

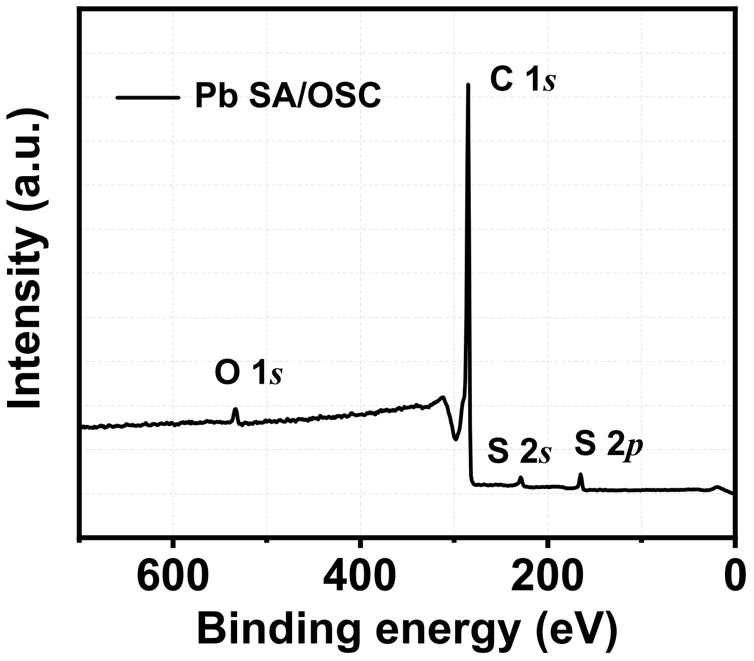

Supplementary Fig. 17 | Composition analysis of Pb SA/OSC. XPS survey spectrum of Pb SA/OSC.

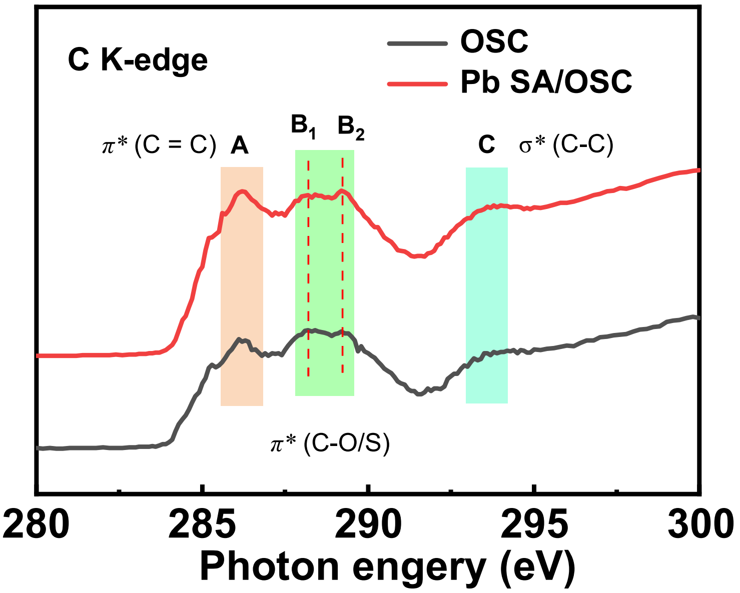

Supplementary Fig. 18 | C K-edge XANES spectra of OSC and Pb SA/OSC. A, B and C attributable to the transition of the 1*s* core electron of carbon into the π^* (C=C), π^* (C–O/S–C), and σ^* (C–C) antibonding states, respectively.

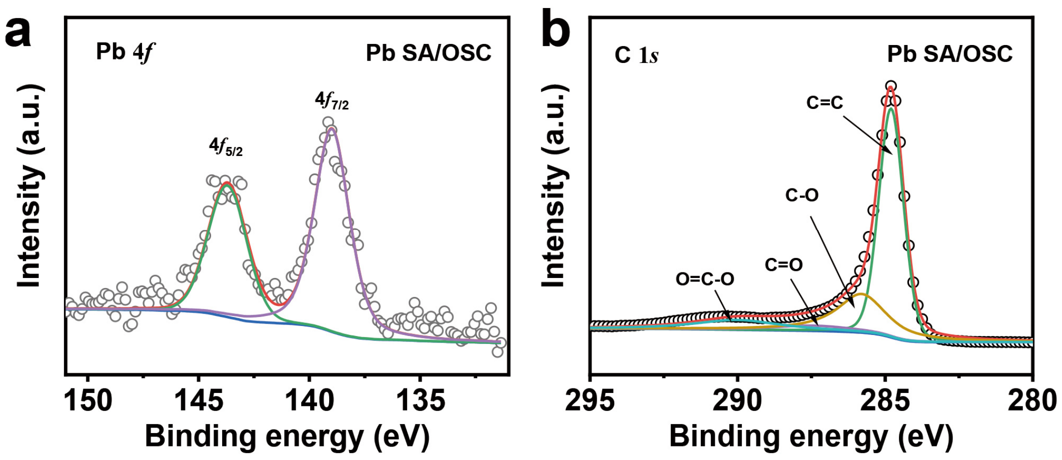

Supplementary Fig. 19 | XPS spectra of Pb SA/OSC. (a, b) Pb 4*f* and C 1*s* XPS spectra of Pb SA/OSC.

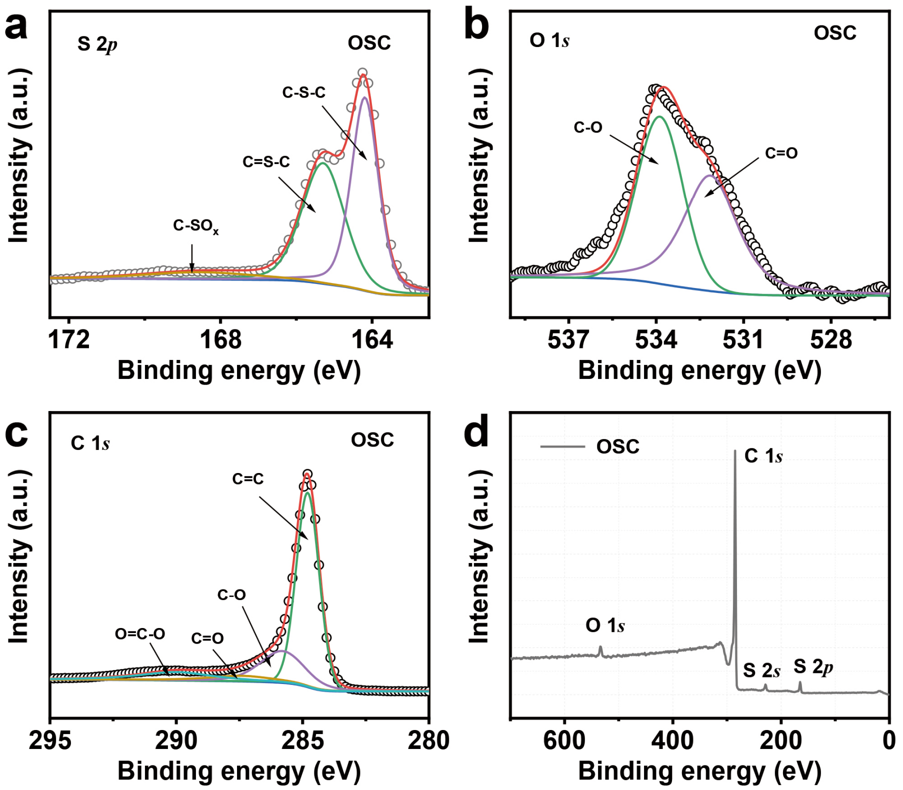

Supplementary Fig. 20 | XPS spectra of OSC. (a-c) The XPS spectra for the S 2*p*, O 1*s* and C 1*s* of OSC. (d) XPS spectrum for the survey scan of OSC.

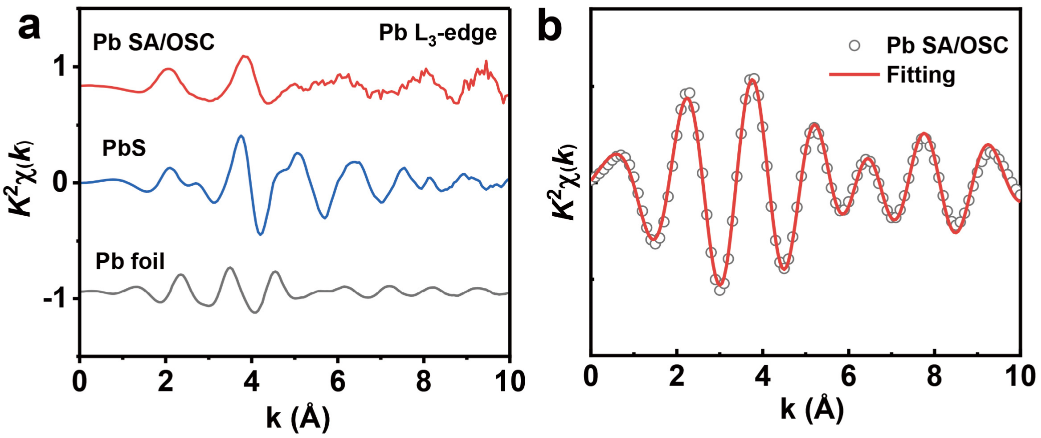

Supplementary Fig. 21 | EXAFS spectra of Pb SA/OSC. (a) EXAFS *k* space curves of Pb SA/OSC, PbS and Pb foil. (b) EXAFS *k* space fitting curves of Pb SA/OSC.

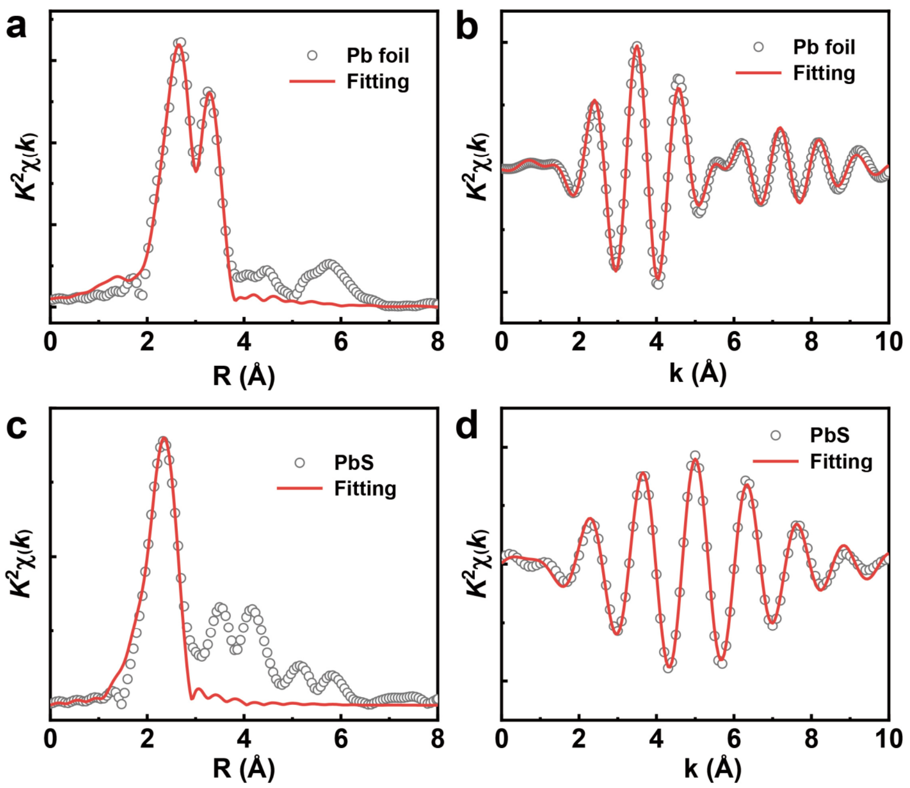

Supplementary Fig. 22 | EXAFS fitting curves of Pb foil and PbS. FT-EXAFS fitting curves (a, c) and EXAFS *k* space fitting curves (b, d) of Pb foil and PbS, respectively.

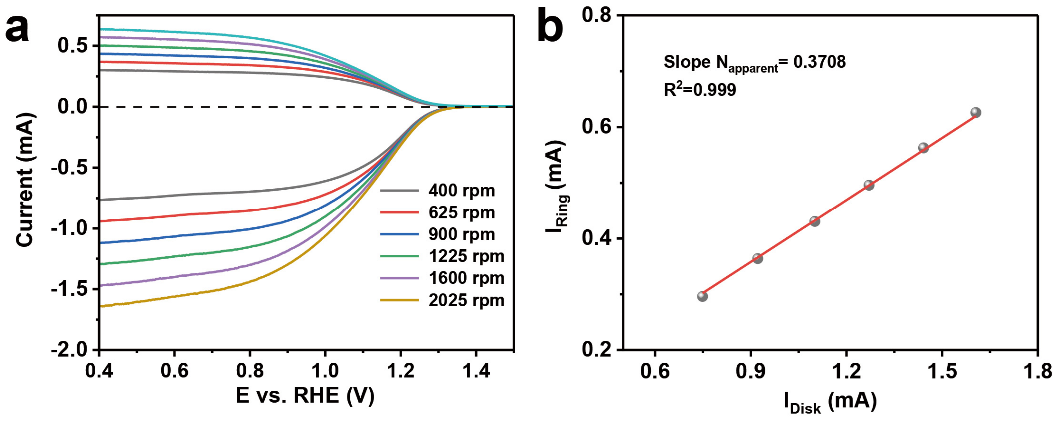

Supplementary Fig. 23 | RRDE collection efficiency calibration. (a) LSV curves recorded on a bare glassy carbon rotation disk electrode (Φ = 5.61 mm) with a Pt ring (Φ = 7.91 mm) in the electrolyte of 0.1 M KOH + 10 mM $K_3Fe(CN)_6$. Sweep rate: 20 mV s^{-1}, Ering = 1.55 V *vs.* RHE. (b) Linear fitting of the diffusion limited current densities recorded on ring and disk electrodes at different rotation speed. The experimental determined apparent collection efficiency (N) is 37.08%, close to the theoretical vale of 37%.

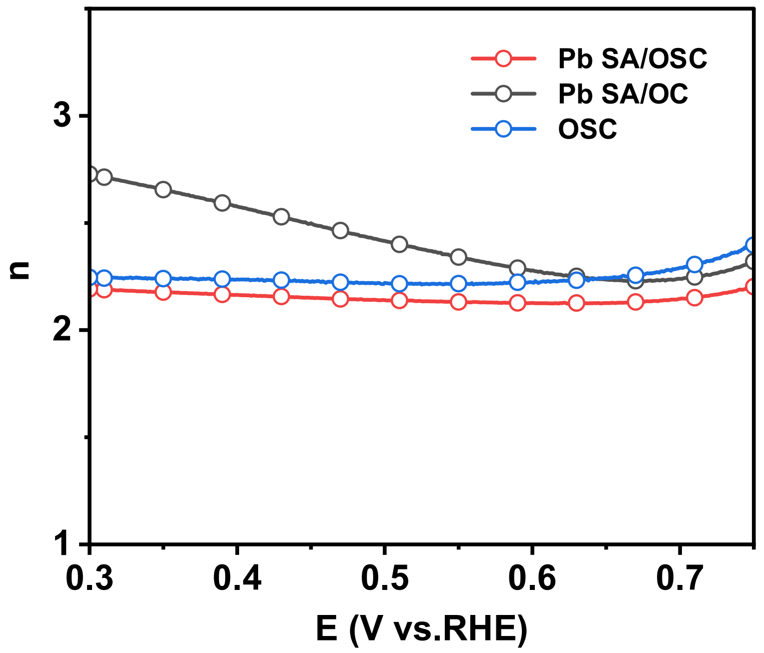

Supplementary Fig. 24 | Calculated electron transfer numbers. Electron transfer numbers (n) of Pb SA/OSC, Pb SA/OC and OSC catalysts.

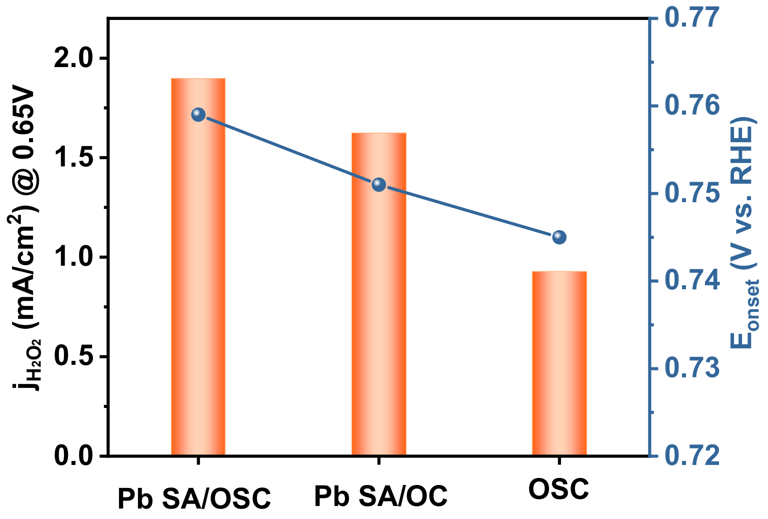

Supplementary Fig. 25 | Comparison of H_2O_2 current density and onset potential. H_2O_2 current density (j_{H2O2}) at 0.65 V and onset potential at j_{Ring} = 0.1 mA cm^{-2} for Pb SA/OSC, Pb SA/OC, and OSC.

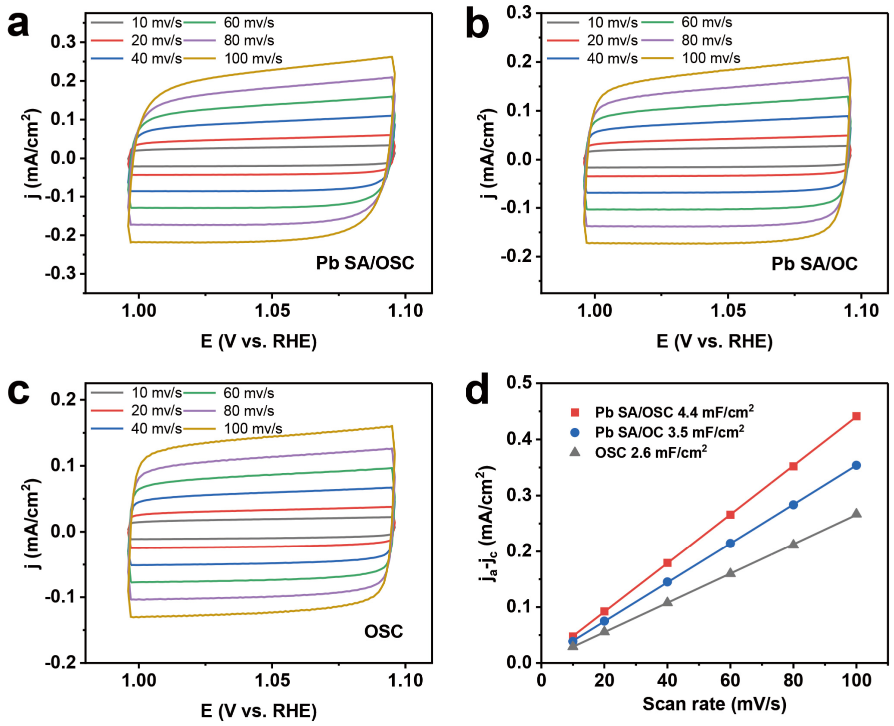

Supplementary Fig. 26 | Non-Faradic double layer capacitance measurements. (a-c) CV curves at different scan rates (10 to 100 mV s^{-1}) for Pb SA/OSC, Pb SA/OC, and OSC in 0.10 M KOH solution, respectively. (d) Capacitive $\Delta j = j_a - j_c$ as a function of the scan rate of catalysts.

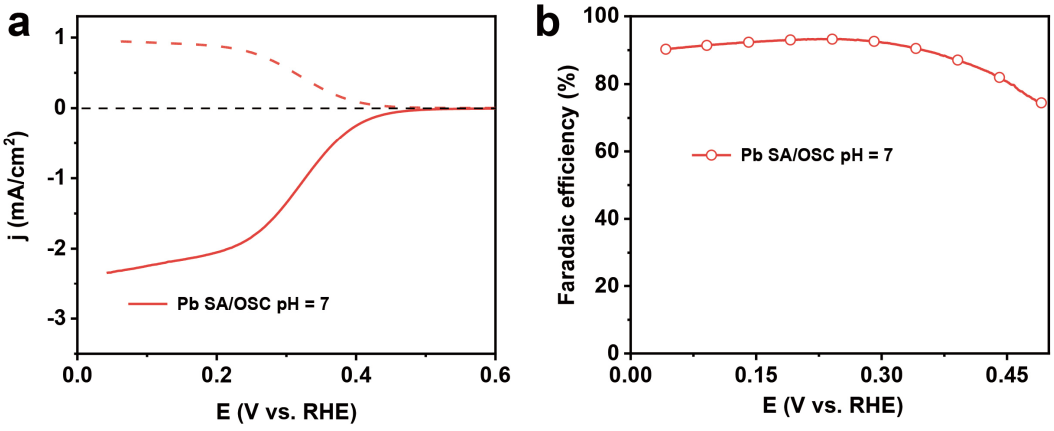

Supplementary Fig. 27 | Two-electron ORR activity and selectivity evaluation of the Pb SA/OSC catalyst in a neutral medium. (a) Electrochemical oxygen reduction polarization curves at the disk electrode (solid lines) together with H_2O_2 detection currents at the ring electrode (dashed lines) for Pb SA/OSC catalyst in 0.1 M PBS electrolyte. (b) Relevant calculated H_2O_2 selectivity based on the RRDE measurements.

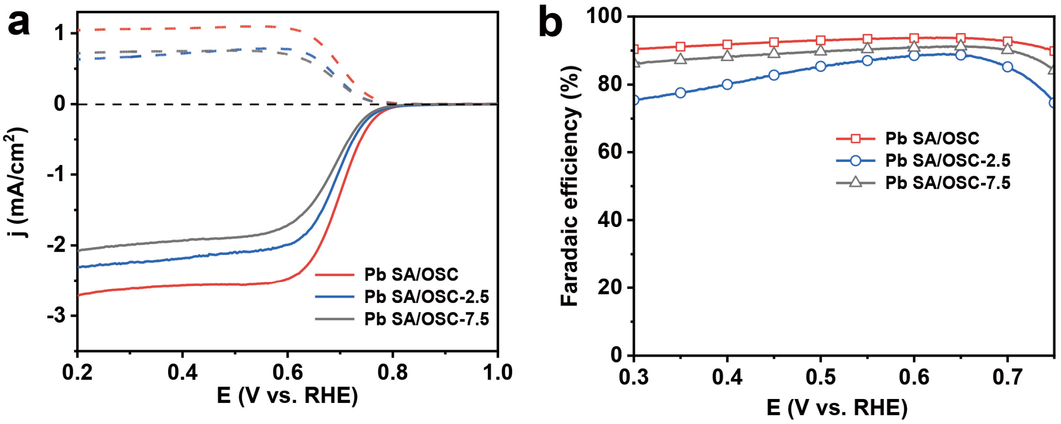

Supplementary Fig. 28 | Two-electron ORR activity and selectivity evaluation of Pb SACs. (a) Electrochemical oxygen reduction polarization curves (solid lines) along with H_2O_2 detection currents (dashed lines) for Pb SACs with different S contents in O_2-saturated 0.10 M KOH electrolyte. (b) H_2O_2 selectivity based on the RRDE measurements.

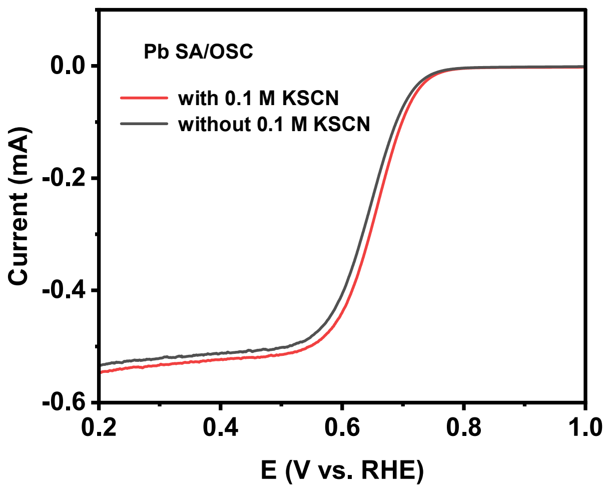

Supplementary Fig. 29 | Thiocyanide poisoning experiments. RDE curves of Pb SA/OSC in O_2-saturated 0.10 M KOH electrolyte with (red lines) and without (black lines) 0.1 M KSCN.

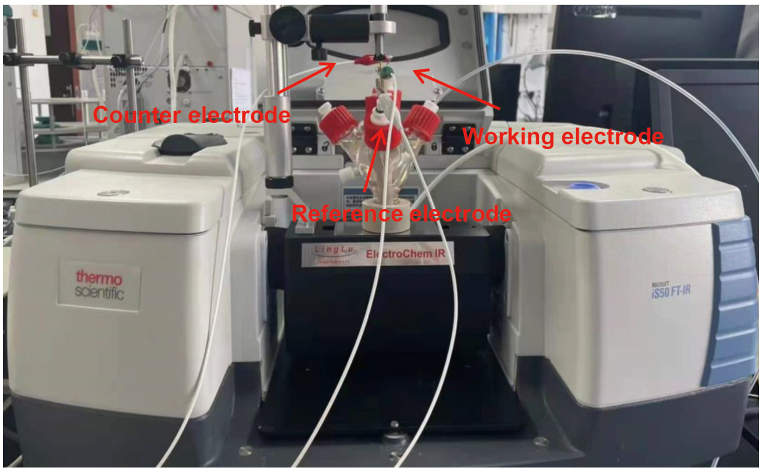

Supplementary Fig. 30 | The detail of the in-situ ATR-SEIRAS experiments. Photograph showing in situ ATR-SEIRAS set-up for electrocatalytic H_2O_2 synthesis.

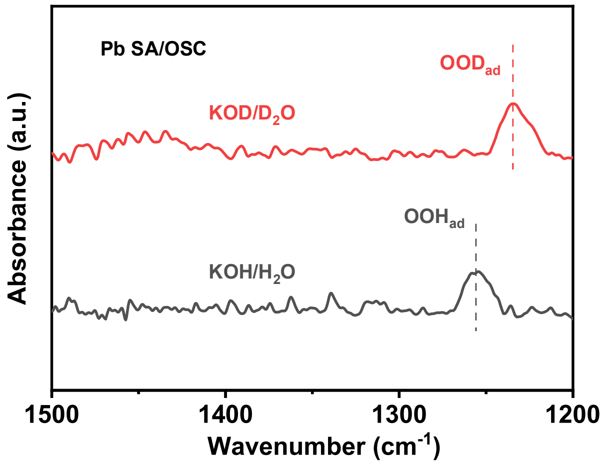

Supplementary Fig. 31 | *In-situ* ATR-SEIRAS isotopic experiments. *In-situ* ATR-SEIRAS spectra collected on the Pb SA/OSC catalyst in an O_2-saturated 0.10 M KOH solution and a deuterated medium.

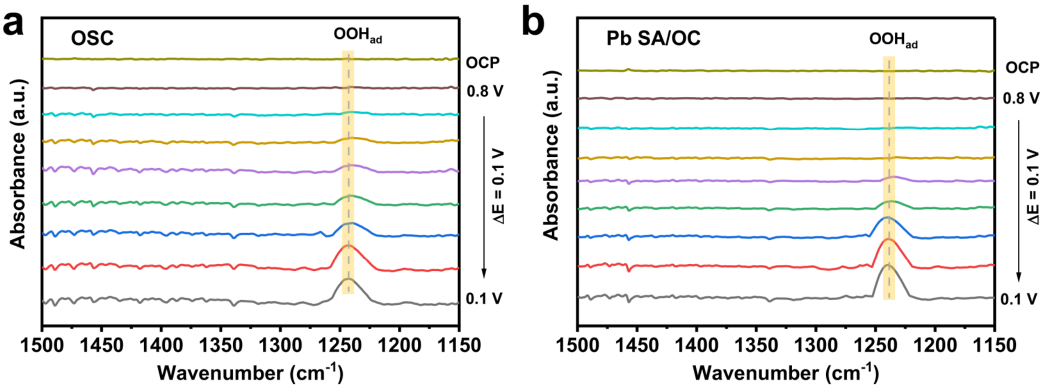

Supplementary Fig. 32 | In-situ ATR-SEIRAS experiments for OSC and Pb SA/OC. (a, b) *In-situ* ATR-SEIRAS spectra collected on the OSC and Pb SA/OC catalysts in O_2-saturated 0.10 M KOH catholyte.

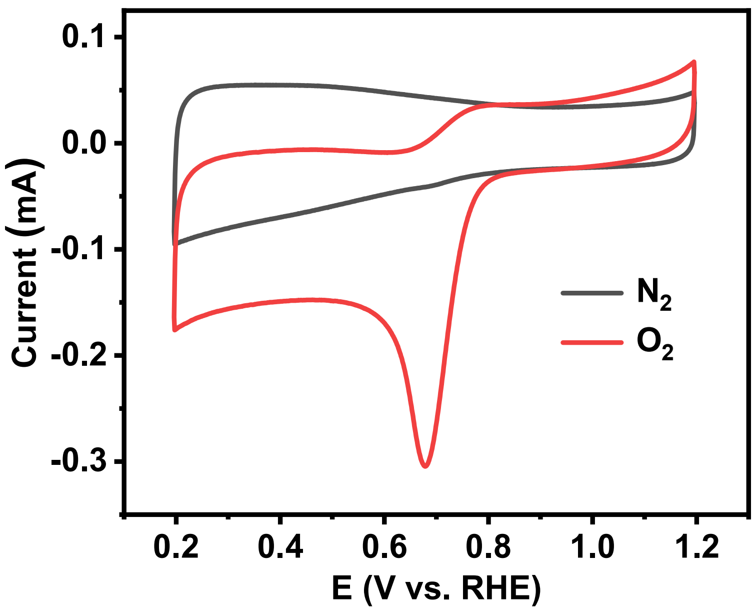

Supplementary Fig. 33 | CV test at different atmosphere. The cyclic voltammetry curves for the Pb SA/OSC catalyst in N_2- and O_2-saturated 0.1 M KOH (scan rate: 50 mV s^{-1}), respectively.

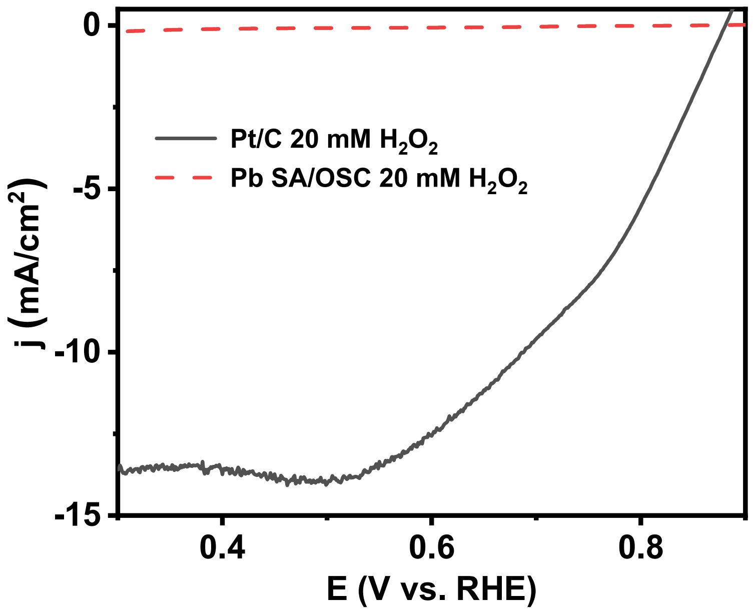

Supplementary Fig. 34 | H_2O_2 electroreduction rate on Pb SA/OSC and Pt/C. Linear sweep voltammograms of Pb SA/OSC and Pt/C in N_2-saturated 0.1 M KOH electrolyte containing 20 mM H_2O_2.

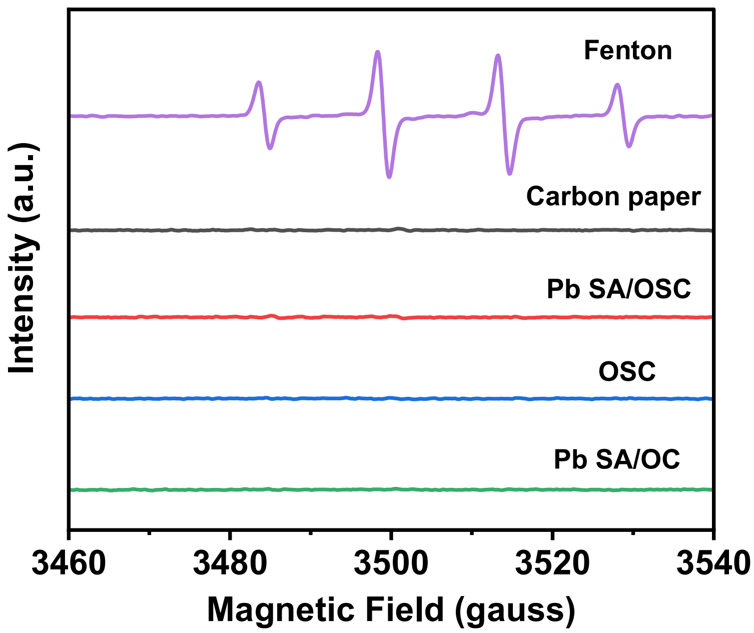

Supplementary Fig. 35 | EPR spectra of carbon paper, Pb SA/OSC, Pb SA/OC, and OSC. EPR spectra were recorded after electrolysis at 0.4 V *vs*. RHE for 3 min, using DMPO as the trapping agent. Fenton reaction condition: [Fe^{2+}] = 1 mM, [H_2O_2] = 4 mM.

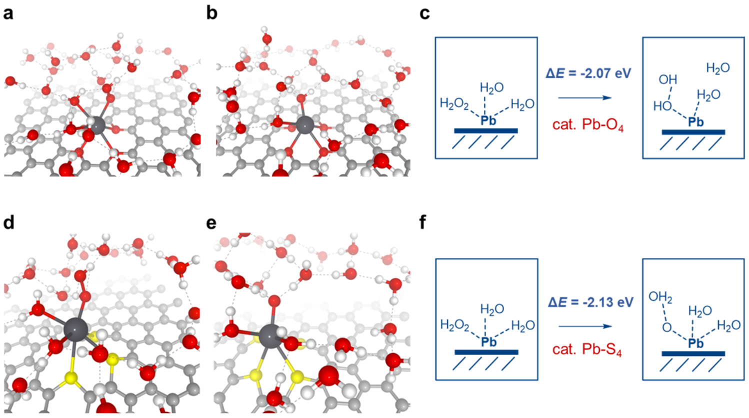

Supplementary Fig. 36 | DFT-optimized geometries of H_2O_2 molecule at the solid–liquid interface. (a-c) Solvated H_2O_2 and the production of *OH intermediates on the PbO_4 catalyst, (d-f) H_2O_2 and the *O intermediates at the PbS_4 catalyst.

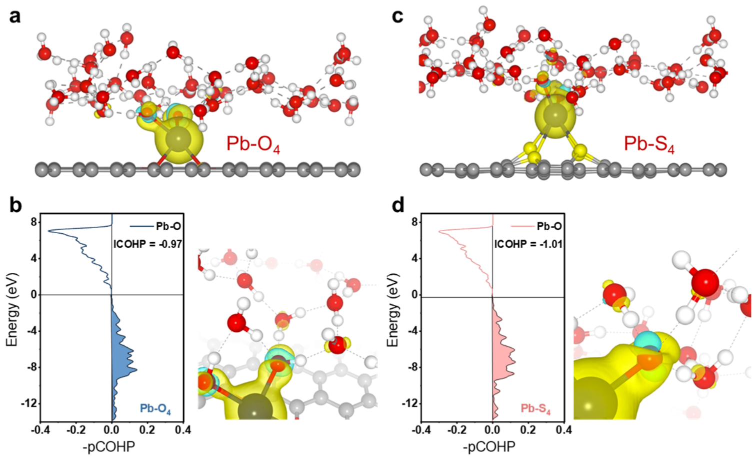

Supplementary Fig. 37 | Charge transfer behavior and molecular bond strength relationship. The charge density differences for H_2O_2 adsorbed on (a) PbO_4 and (c) PbS_4. The yellow color in the representation signifies a high electron density, while the blue color indicates scarce electron density. Electron density difference maps of 0.008 $e^{-}/bohr^3$. The crystal orbital Hamilton populations (COHP) of (b) *OH adsorbed on PbO_4 and (d)*O adsorbed on PbS_4 after H_2O_2 activation.

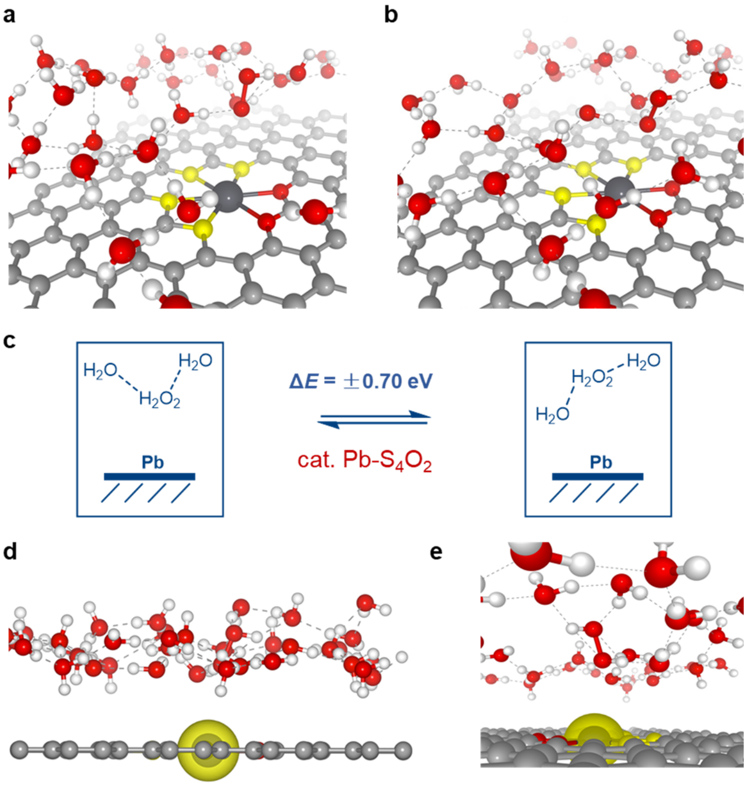

Supplementary Fig. 38 | DFT-optimized geometries of H_2O_2 molecule at the solid–liquid interface. (a-c) Solvated H_2O_2 with two different adsorption structures on the PbS_4O_2 catalyst, (d, e) Calculated charge density differences for H_2O_2 on PbS_4 O_2 (isosurface value=0.008 $e^-/bohr^3$).

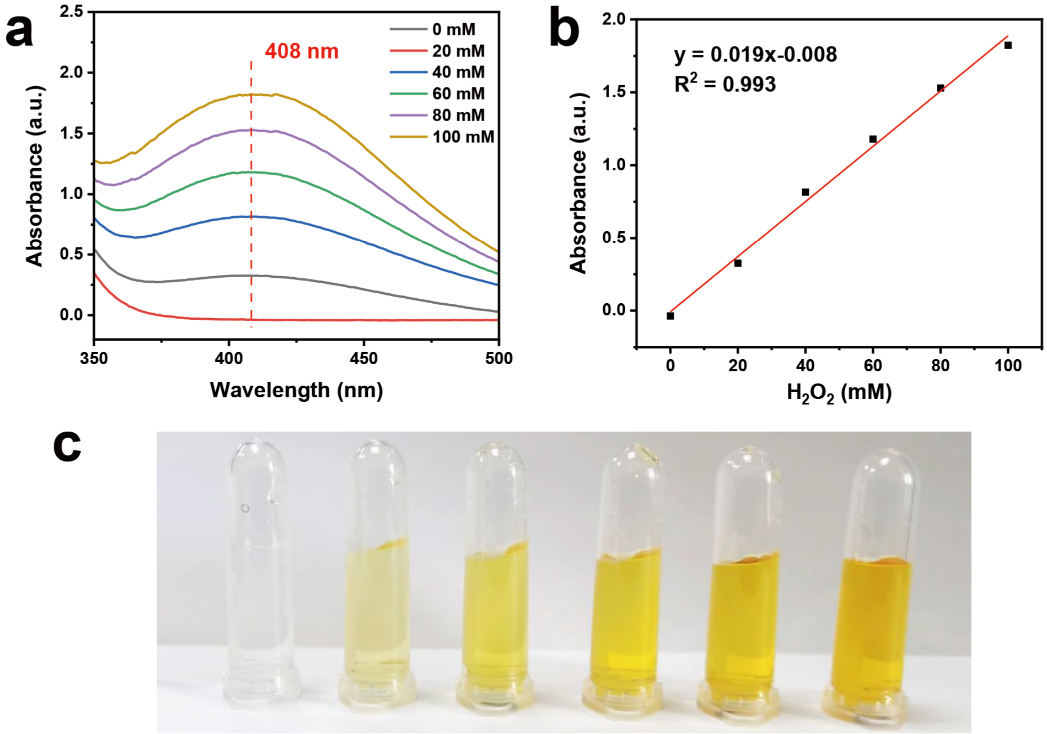

Supplementary Fig. 39 | Colorimetric quantification of H_2O_2 content. The commercial H_2O_2 solution (30 wt%) was diluted with DI water to the desired concentration. The accurate H_2O_2 concentration was obtained by potassium permanganate titration method. (a) Absorption spectra of $H_2[Ti(O_2)(SO_4)_2]$ solution obtained by adding a series of H_2O_2 solutions with known concentration to 2 wt% $TiOSO_4$ in 2 M H_2SO_4. (c) The corresponding digital photographs. (b) Linear calibration curve based on the peak absorbance at 408 nm.

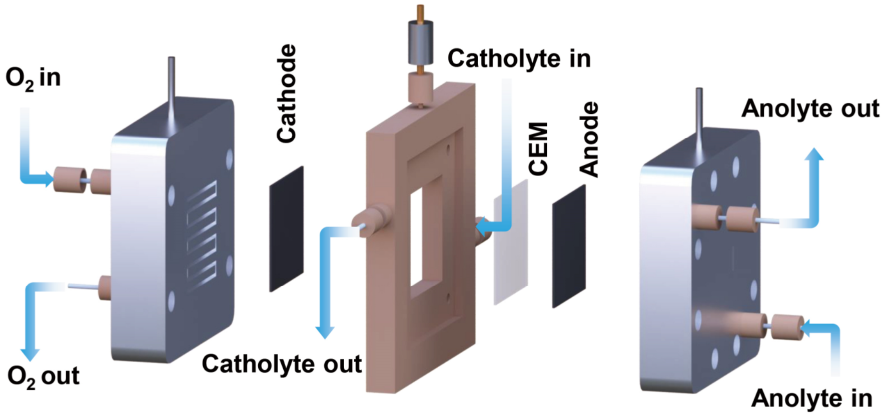

Supplementary Fig. 40 | Schematic illustration of the flow-cell setup. H_2O_2 is produced by O_2 reduction reaction at the cathode and flowed out with the catholyte flow.

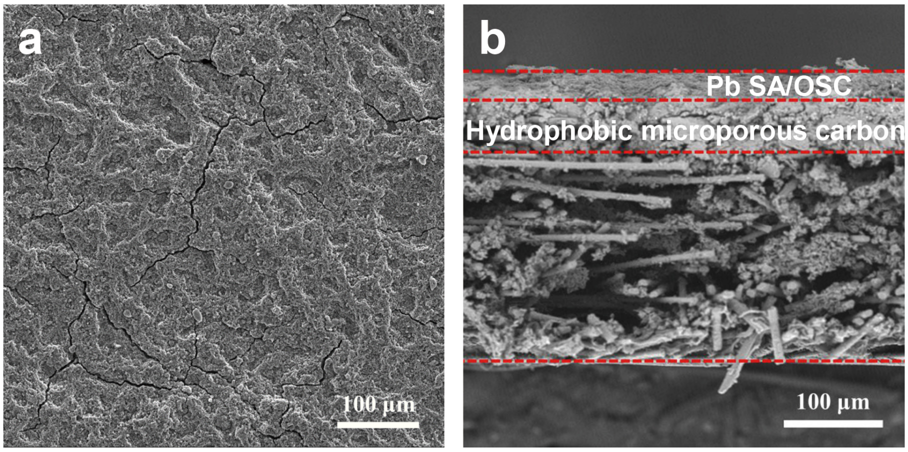

Supplementary Fig. 41 | Morphology characterizations of the prepared sample on the carbon-based gas diffusion layer (GDL). SEM images of Pb SA/OSC on the GDL (a, b).

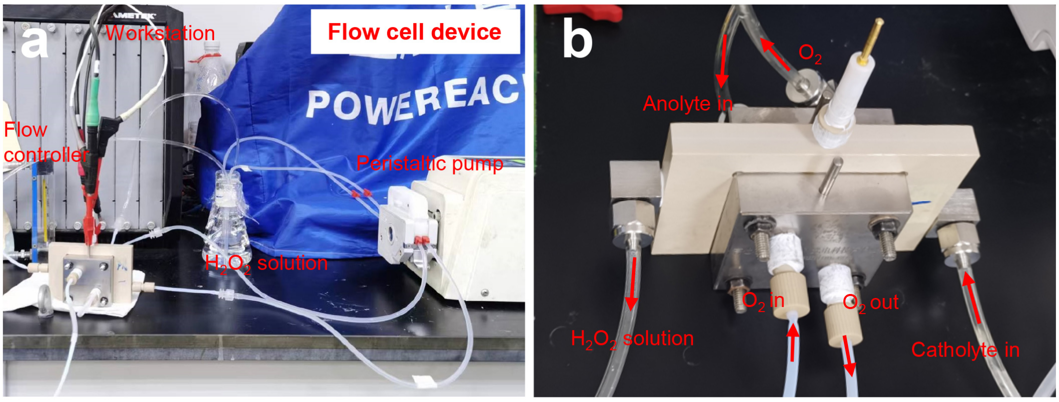

Supplementary Fig. 42 | Digital photograph of a flow cell setup for the electrochemical 2e⁻ ORR measurements. (a) An overview of the system approach used in the experiments. (b) Exploded view of the assembled cell.

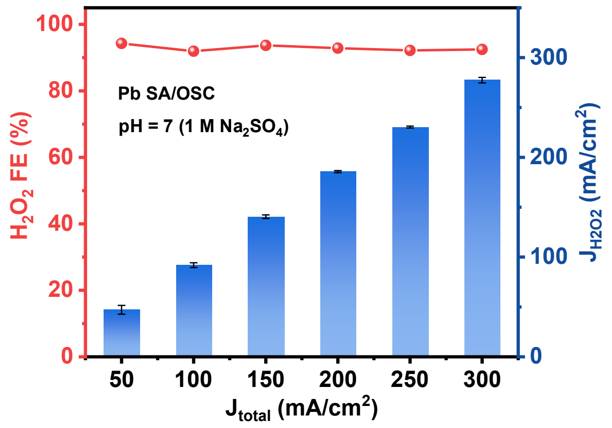

Supplementary Fig. 43 | H_2O_2 Faradaic efficiencies and J_{H2O2} of Pb SA/OSC using gas diffusion electrodes in a neutral medium. Error bars correspond to the standard deviation of three independent measurements.

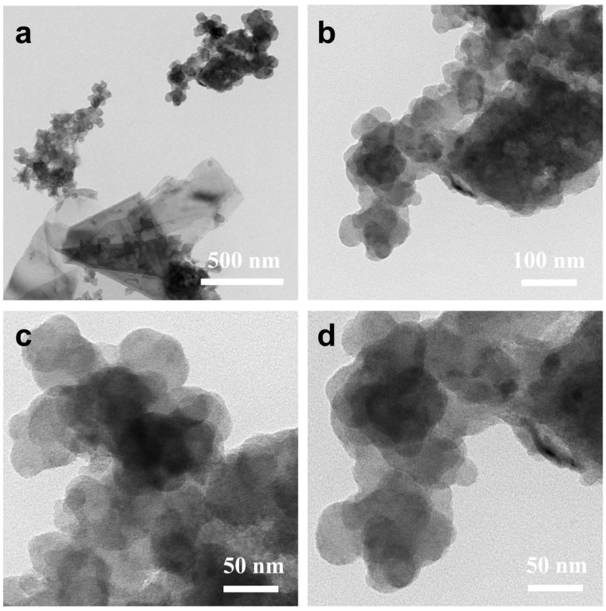

Supplementary Fig. 44 | Morphology characterizations of Pb SA/OSC after the stability test. (a-d) TEM images at different magnifications of Pb SA/OSC obtained from the GDE electrode after the stability test.

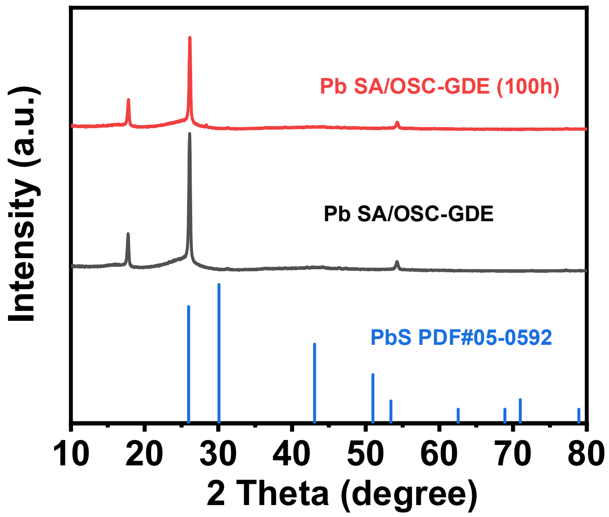

Supplementary Fig. 45 | Structural characterizations of Pb SA/OSC after the stability test. XRD patterns of Pb SA/OSC-GDE before and after the stability test.

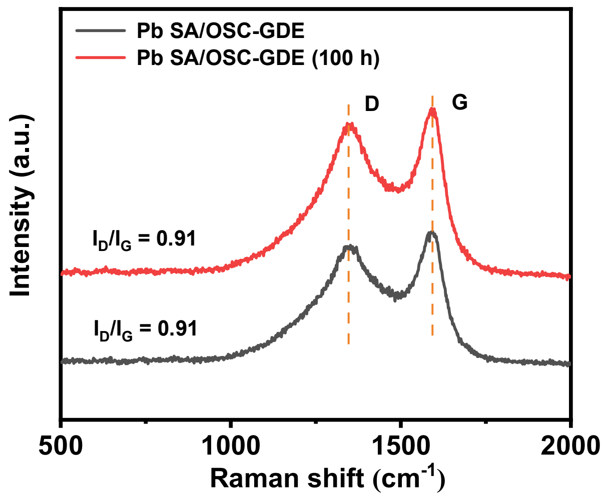

Supplementary Fig. 46 | Raman spectra of Pb SA/OSC-GDE before and after the stability test. The intensity ratios (I_D/I_G) of the D-band to G-band were calculated.

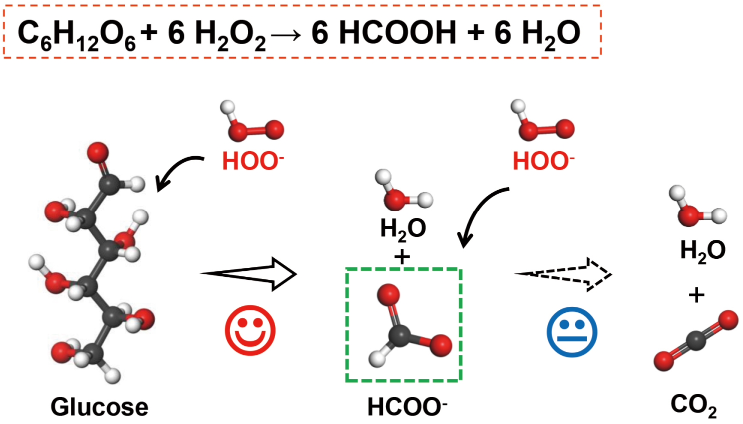

Supplementary Fig. 47 | Schematic illustration of glucose oxidation for the production of formic acid. The alkali present in the electrolyte plays a pivotal role in preventing the excessive of the produced HCOOH into CO_2 and H_2O.

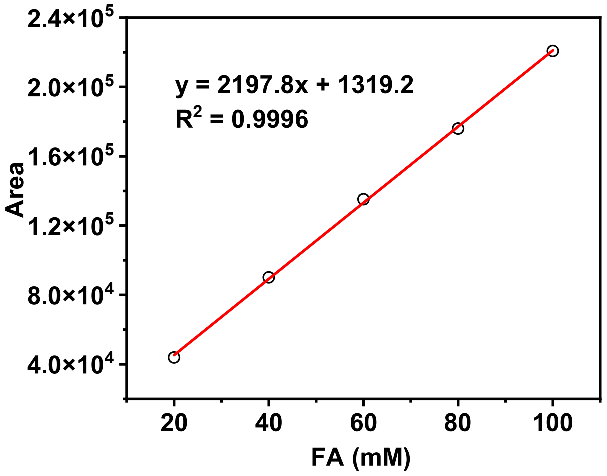

Supplementary Fig. 48 | The determination of the produced formic acid. Linear calibration curve of formic acid concentration (20, 40 ,60, 80 and 100 mM) based on HPLC results.

Supplementary Table 1. Interatomic distances of Pb–S and Pb–O of different optimized catalysts.

Model		Pb–S (Å)			Pb–O (Å)			
PbO_4	/	/	/	/	2.56	2.60	2.56	2.60
PbS_1O_3	2.87	/	/	/	2.57	2.72	2.83	/
PbS_2O_2	2.94	2.96	/	/	2.72	2.73	/	/
PbS_3O_1	2.91	3.20	2.80	/	3.13	/	/	/
PbS_3O_1-f	3.20	2.97	2.96	/	2.78	/	/	/
PbS_4	2.98	3.08	2.95	3.02	/	/	/	/
PbS_4-f	3.09	3.13	3.02	2.99	/	/	/	/
PbS_4O_2	2.45	2.60	2.45	2.60	2.39	2.40	/	/

Supplementary Table 2. Interatomic distances of Pb–OOH and O–O in *OOH on the Pb site of different catalysts.

Distance	PbS_4	PbS_3O_1	PbS_2O_2	PbS_1O_3	PbO_4	PbS_4O_2
Pb–OOH	2.15	2.16	2.22	2.16	2.18	2.39
O–O	1.50	1.47	1.49	1.48	1.46	1.44

Supplementary Table 3. Free energies *G* (eV) for the adsorbates on the Pb site of different catalysts at T = 298 K.

Adsorbate	PbS_4	PbS_3O_1	PbS_4O_2
*OOH	3.27	3.32	4.21
*O	0.67	1.33	2.0
*OH	-0.25	-0.12	0.53

Supplementary Table 4. Contributions to the free energy for reference molecules. A fugacity of 3534 Pa for $H_2O(l)$ according to the vapour pressure of water and the vapour-liquid equilibrium of water at T = 298 K.

Molecule	ZPE (eV)	*E* (eV)	G_{corr} (eV)	*G* (eV)
$H_2O(l)$	0.57	-14.22	0.00	-14.22
$H_2(g)$	0.27	-6.76	-0.04	-6.80

Supplementary Table 5. Vibrational frequencies used to determine the zero-point energy correction (ZPE) for adsorbates (*OOH, *O and *OH) at the Pb sites of various catalysts at T = 298 K.

Catalyst	PbS_3O_1-f	PbS_4-f	PbO_4	PbS_1O_3	PbS_2O_2	PbS_3O_1	PbS_4	PbS_4O_2
υ_{*OOH} (cm^{-1})	3513.03<br>1344.22<br>802.28<br>605.9<br>399.85<br>112.17	3631.8<br>1303.86<br>802.65<br>469.97<br>368.07<br>238.39	3447.59<br>1302.07<br>1252.33<br>549.21<br>283.12<br>108.35	3634.48<br>1329.53<br>809.83<br>429.45<br>316.14<br>82.22	3639.69<br>1267.21<br>830.03<br>454.92<br>250.86<br>72.16	3637.03<br>1334.13<br>862.5<br>454.02<br>359.68<br>71.06	3431.78<br>1329.1<br>800.33<br>586.01<br>373.91<br>108.33	3557.06<br>1315.82<br>878.38<br>433.61<br>254.9<br>136.14
$\Sigma\upsilon_{*OOH}$ (cm^{-1})	6777.45	6932.61	6942.68	6601.66	6514.88	6718.42	6629.46	6575.91
ZPE_{*OOH} (eV)	0.42	0.43	0.43	0.41	0.4	0.42	0.41	0.41
υ_{*O} (cm^{-1})	684.83<br>164.24	697.2	1255.52	661.8	770.21<br>181.61	597.57	693.21<br>35.58	614.14
$\Sigma\upsilon_{*O}$ (cm^{-1})	849.08	697.2	1255.52	661.8	951.82	597.57	728.79	614.14
ZPE_{*O} (eV)	0.05	0.04	0.08	0.04	0.06	0.04	0.05	0.04
υ_{*OH} (cm^{-1})	3742.23<br>646.65<br>416.27<br>371.09	3825.75<br>660.99<br>357.69<br>300.62	3829.68<br>626.27<br>403.26<br>255.75	3762.94<br>839.69<br>354.47<br>222.42	3807.04<br>555.35<br>431.9<br>197.26	3474.01<br>703.55<br>515.08<br>378.87	3718.68<br>686.01<br>411.1<br>378.35	3648.81<br>569.01<br>362.27
$\Sigma\upsilon_{*OH}$ (cm^{-1})	5176.24	5145.05	5114.96	5179.52	4991.54	5071.51	5194.15	4580.1
ZPE_{*O} (eV)	0.32	0.32	0.32	0.32	0.31	0.31	0.32	0.28

Supplementary Table 6. Structural parameters extracted from the Pb L_3-edge EXAFS fitting.

Sample	Scattering pair	CN	R (Å)	σ^2 (10^{-3}Å^2)	ΔE_0 (eV)	R factor
Pb SA/OSC	Pb-S	3.5	2.75	16.3	-11	0.016
	Pb-O	1.7	2.26	3.38	-4.1	
PbS	Pb-S	6.0	2.93	14.4	-0.95	0.014
Pb foil	Pb-Pb	12.0	3.38	22.5	-7.3	0.011

S_0^2 is the amplitude reduction factor; CN is the coordination number; R is interatomic distance (the bond length between central atoms and surrounding coordination atoms); σ^2 is Debye-Waller factor (a measure of thermal and static disorder in absorber-scatterer distances); ΔE_0 is edge-energy shift (the difference between the zero kinetic energy value of the sample and that of the theoretical model). R factor is used to value the goodness of the fitting.

Error bounds that characterize the structural parameters obtained by EXAFS spectroscopy were estimated as N ± 20%; R ± 1%; σ^2 ± 20%; ΔE_0 ± 20%.

Supplementary Table 7. Comparison of the electrochemical O_2-to-H_2O_2 performance on current state of-the-art catalysts at high current densities.

Catalyst	FE (%)	J (mA cm^{-2})	Productivity (mmol cm^{-2} h^{-1})	Stability	Ref.
**Pb SA/OSC**	**96.6**<br>**97.1**<br>**92.7**	**200**<br>**300**<br>**400**	**3.6**<br>**5.4**<br>**6.9**	**50 mA/cm^2 for 100 h**	**This work**
N_4-Ni_1-O_2/OCNTs	96.1<br>90.8<br>87.6	200<br>300<br>350	3.7<br>5.1<br>5.7	200 mA/cm^2 for 24 h	1
Graphene array	94<br>81	100<br>200	1.8<br>3	100 mA/cm^2 for 16 h	2
N_3-In_1-S_1	90	110	1.29	0.3V for 12 h	3
N-doped carbon	99	100	1.7	100 mA/cm^2 for 200 h	4
NiN_2O_2/C	97	65	1.18	70 mA/cm^2 for 8 h	5
Co-N-C	95.6	50	0.9	/	6
B-C	85.1	300	3.17	200 mA/cm^2 for 30 h	7
molecular Co–N–C	70	411	5.38	100 mA/cm^2 for 48 h	8
NADE	82<br>64.8	60<br>240	0.9<br>2	/	9
CoN_4/VG	70	21	0.304	210 mA/cm^2 for 6 h	10
Pt-S-C	70	10	0.097	10 mA/cm^2 for 8.3 h	11
CoPc-OCNT	98	300	5.48	200 mA/cm^2 for 30 h	12
Sb-NSCF	80	50	0.746	50 mA/cm^2 for 75 h	13
HCNFs	95	13	0.23	/	14
Co-POC-O	64	10	0.12	/	15
Co-N-C(2)	50	50	0.4	70 mA/cm^2 for 6 h	16
OCNS900	90	50	0.8	50 mA/cm^2 for 11 h	17
N-FLG-8	99.8	36	0.7	20 mA/cm^2 for 50 h	18
C-O	95	15	0.3	/	19

References

1. Xiao, C. *et al.* Super-coordinated nickel $N_4Ni_1O_2$ site single-atom catalyst for selective H_2O_2 electrosynthesis at high current densities. *Angew. Chem., Int. Ed.* **61**, e202206544 (2022).
2. Wang, Y. *et al.* Vertical graphene array for efficient electrocatalytic reduction of oxygen to hydrogen peroxide. *Nano Energy* **96**, 107046 (2022).
3. Zhang, E. *et al.* Engineering the local atomic environments of indium single-atom catalysts for efficient electrochemical production of hydrogen peroxide. *Angew. Chem., Int. Ed.* **61**, e202117347 (2022).
4. Cao, P. *et al.* Durable and selective electrochemical H_2O_2 synthesis under a large current enabled by the cathode with highly hydrophobic three-phase architecture. *ACS Catal.* **11**, 13797-13808 (2021).
5. Wang, Y. *et al.* High-efficiency oxygen reduction to hydrogen peroxide catalyzed by nickel single-atom catalysts with tetradentate N_2O_2 coordination in a three-phase flow cell. *Angew. Chem., Int. Ed.* **59**, 13057-13062 (2020).
6. Zhao, Q. *et al.* Approaching a high-rate and sustainable production of hydrogen peroxide: oxygen reduction on Co–N–C single-atom electrocatalysts in simulated seawater. *Energy Environ. Sci.* **14**, 5444-5456 (2021).
7. Xia, Y. *et al.* Highly active and selective oxygen reduction to H_2O_2 on boron-doped carbon for high production rates. *Nat. Commun.* **12**, 4225 (2021).
8. Liu, C. *et al.* Heterogeneous molecular Co–N–C catalysts for efficient electrochemical H_2O_2 synthesis. *Energy Environ. Sci.* **16**, 446-459 (2023).
9. Zhang, Q. *et al.* Highly efficient electrosynthesis of hydrogen peroxide on a superhydrophobic three-phase interface by natural air diffusion. *Nat. Commun.* **11**, 1731 (2020).
10. Lin, Z. *et al.* Atomic Co decorated free-standing graphene electrode assembly for efficient hydrogen peroxide production in acid. *Energy Environ. Sci.* **15**, 1172-1182 (2022).
11. Zhao, J. *et al.* Manipulating the oxygen reduction reaction pathway on Pt-coordinated motifs. *Nat. Commun.* **13**, 685 (2022).
12. Cao, P. *et al.* Metal single-site catalyst design for electrocatalytic production of hydrogen peroxide at industrial-relevant currents. *Nat. Commun.* **14**, 172 (2023).
13. Yan, M. *et al.* Sb_2S_3-templated synthesis of sulfur-doped Sb-N-C with hierarchical

architecture and high metal loading for H_2O_2 electrosynthesis. *Nat. Commun.* **14**, 368 (2023).

14. Dong, K. *et al.* Honeycomb carbon nanofibers: A superhydrophilic O_2-entrapping electrocatalyst enables ultrahigh mass activity for the two-electron oxygen reduction reaction. *Angew. Chem., Int. Ed.* **60**, 10583-10587 (2021).
15. Li, B.-Q., Zhao, C.-X., Liu, J.-N. & Zhang, Q. Electrosynthesis of hydrogen peroxide synergistically catalyzed by atomic Co–N_x–C sites and oxygen functional groups in noble-metal-free electrocatalysts. *Adv. Mater.* **31**, 1808173 (2019).
16. Sun, Y. *et al.* Activity–selectivity trends in the electrochemical production of hydrogen peroxide over single-site metal–nitrogen–carbon catalysts. *J. Am. Chem. Soc.* **141**, 12372-12381 (2019).
17. Chen, S. *et al.* Chemical identification of catalytically active sites on oxygen-doped carbon nanosheet to decipher the high activity for electro-synthesis hydrogen peroxide. *Angew. Chem., Int. Ed.* **60**, 16607-16614 (2021).
18. Li, L. *et al.* Tailoring selectivity of electrochemical hydrogen peroxide generation by tunable pyrrolic-nitrogen-carbon. *Adv. Energy Mater.* **10**, 2000789 (2020).
19. Xia, C., Xia, Y., Zhu, P., Fan, L. & Wang, H. Direct electrosynthesis of pure aqueous H_2O_2 solutions up to 20% by weight using a solid electrolyte. *Science* **366**, 226-231 (2019).
